# Highly Sensitive Microstructure-Based Flexible Pressure Sensor for Quantitative Evaluation of Motor Function Recovery after Spinal Cord Injury

**DOI:** 10.3390/s19214673

**Published:** 2019-10-28

**Authors:** Dan Yang, Wei Yang, Lianhui Li, Kai Zhou, Mingming Hao, Xingyu Feng, Ting Zhang, Yaobo Liu

**Affiliations:** 1 Jiangsu Key Laboratory of Neuropsychiatric Diseases and Institute of Neuroscience, Soochow University, Suzhou 215123, China; stefanie111@126.com (D.Y.); weiyang7vivi@163.com (W.Y.); 20164254004@stu.suda.edu.cn (K.Z.); fxy981118@126.com (X.F.); 2 *i*-Lab., Key Laboratory of Multifunctional Nanomaterials and Smart Systems, Suzhou Institute of Nano-tech and Nano-bionics, Chinese Academy of Sciences, Suzhou 215123, China; lhli2015@sinano.ac.cn (L.L.); mmhao2016@sinano.ac.cn (M.H.); 3 Department of Anatomy, School of Basic Medical Science, Guizhou Medical University, Guiyang 550025, China

**Keywords:** highly sensitive, flexible pressure sensor, quantitative evaluation, spinal cord injury

## Abstract

Behavioral assessment, such as systematic scoring or biomechanical measurement, is often used to evaluate the extent of the damage and the degree of recovery after spinal cord injury. However, the use of these methods in standardized evaluation is limited because they are subjective and require complex test systems to implement. Here, we report a novel, flexible, microstructure-based pressure sensor and demonstrate its superior sensitivity (235.12 kPa^−1^ for 5.5~135 Pa and 2.24 kPa^−1^ for 0.6~25 kPa), good waterproofness, fast response and recovery times (response time: 8 ms, recovery time: 12 ms), stable response over 8000 loading/unloading cycles, and wide sensing range. These features readily allow the sensor to be comfortably attached to the hindlimbs of mice for full-range, real-time detection of their behavior, such as crawling and swimming, helping to realize quantitative evaluation of animal motor function recovery after spinal cord injury.

## 1. Introduction

Severe spinal cord injury (SCI) interrupts nearly all brain projections to lumbar circuits, resulting in motor function loss and paralysis [1,2]. Therefore, behavioral monitoring of hindlimb motor function is particularly important after injury, allowing one to evaluate not only motor function recovery but also the integrity of the sensorimotor neural circuit [3,4]. Various readouts have been used to evaluate locomotor deficits, such as endpoint measures, biomechanical measures, and electromyography (EMG). However, these methods are accompanied by several restrictions. For example, scoring is the most commonly used method in behavioral assessment [5,6,7], but it is not sensitive enough to recognize subtle movements and is not suitable for all types of spinal cord injuries. Gait kinematics [8,9,10] requires a whole set of weight-bearing devices and treadmill systems, which render it difficult for some laboratories to perform. Compared with other types of injuries, severe SCI, especially the complete transection injury of the spinal cord, is associated with poor behavioral recovery. Although EMG can be used to observe the recovery of hindlimb function, the implantation of many electrodes damages the hindlimb muscle. Additionally, to operate in a living body, the electrodes must have their wires implanted under the skin, making the site prone to infection. Therefore, there is a high demand for a sensitive, noninvasive device that can effectively monitor the motor function recovery of the hindlimbs after severe SCI.

Established scoring systems are usually developed for specific types of injuries. Highly sensitive biomechanical measurements or electromyographic recordings usually focus on some very specific aspects of movement, such as joint angles [8,9]. However, in the situation of paralysis of the lower limbs after severe spinal cord injury, we cannot see that animal lower limbs can support body weight or stand. Therefore, with some limitations, many evaluation methods are not applicable. Especially for the recovery of motor function caused by the remodeling of local nerve circuits after spinal cord injury [11], the recovery of motor function is very slight in the early stage after injury [12]. However, this objective and specific detection method for this recovery are lack, which results in the resistance of early work. In addition, it is more difficult to analyze various behaviors that rodents show according to the environment, such as normal walking or swimming. The nerve circuits involved in the completion are not the same, and different manifestations also reflect the different recovery of nerve circuits [13]. Therefore, we urgently need an evaluation method with high sensitivity and excluding the influence of subjective factors to improve the detection of motor function after spinal cord injury.

Flexible wearable sensors that can measure and quantify electronic signals are attracting great attention because they have unique characteristics, such as ultrathinness, a low modulus, lightweight, and high flexibility [14,15,16]. These flexible sensors can be conformally attached to the irregular surfaces of organs or skin, providing a new opportunity for noninvasive behavior monitoring [17,18,19]. To the best of our knowledge, despite great progress in flexible wearable sensors, a design for a sensitive, flexible sensor with good conformability to realize quantitative evaluation of animal motor function recovery after SCI has not yet been reported. 

**Figure 1:**
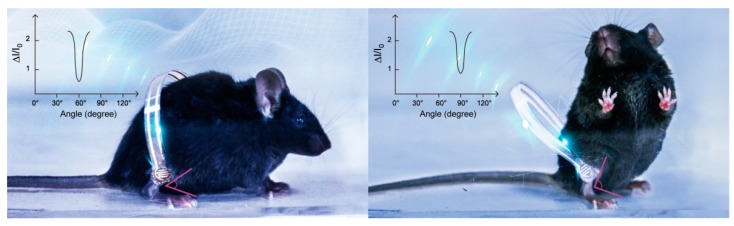
Schematic diagram of the flexible pressure sensor attached to the legs of mice for full-range and real-time detection of their behaviors.

The purpose of our experiment is to report a novel, flexible, microstructure-based pressure sensor and demonstrate its outstanding sensing performance in the quantitative evaluation of animal motor function recovery after SCI (demonstration shown in Figure 1). By choosing two severe models of spinal cord injury, complete spinal transection, and unilateral thoracic spinal cord injury, we compared the recovery of lower limbs in the early stage of spinal cord injury (less than two weeks), including the intensity and frequency of lower extremity activity. Through this method, we hope to clarify the feasibility of such a flexible pressure sensor, used in different environments, for early assessment of lower limb activity in spinal cord injury. It provides an objective and comprehensive evaluation method for our subsequent research on specific nerve circuits.

## 2. Materials and Methods

### 2.1. Materials and Animals

Chlorotrimethylsilane was purchased from Shanghai Titan Scientific Co., Ltd., CP. Polydimethylsiloxane (PDMS) Sylgard 184 was bought from Dow Corning. Single-walled carbon nanotubes (SWCNTs) were purchased from Nanjing XFNANO Materials Tech Co., Ltd., Nanjing, China. All procedures were performed in accordance with the Institute of Neuroscience (Soochow University) guidelines for the use of experimental animals and were approved by the Institutional Animal Care and Use Committee at Soochow University. All mice were on a C57BL/6J background and were kept in the specific-pathogen-free animal facility at Soochow University. The mice were 2–3 months old, and both sexes were included. The mice were housed in groups of five per cage in a 12:12 h light:dark cycle with food and water ad libitum.

### 2.2. Preparation of Flexible Pressure Sensors

The patterned Si mold was dipped in the mold release agent chlorotrimethylsilane to facilitate the release of the cured polydimethylsiloxane (PDMS) from Si mold. A 10:1 mixture of PDMS (Sylgard 184, Dow Corning, Midland, MI, USA) was spin-coated on the Si mold to obtain a flexible PDMS substrate with a pyramid pattern, and the thickness of the patterned PDMS was controlled by the spin coating speed (1000 rpm). Next, the Si mold covered with PDMS was cured at 80 °C for 2 h. Subsequently, the patterned PDMS was placed on a hot plate (IKA, HP4) at 150 °C and sprayed with an SWCNT/ethanol dispersion (0.5 mL, 0.1 mg/mL, ultrasonicated for 20 min) to form a conductive layer. A sealant was used to attach the conductive film with a pyramid microstructure to a thin circular PET substrate with double-helix Ag electrodes prepared by screen printing. The diameter of the pressure sensitive region is 1 cm.

### 2.3. Surgery and Animal Care

Complete spinal transection or unilateral thoracic SCI was carried out at T9 by modifying previously described methods. After the surgery, the muscle layers, fascia, and skin were sequentially sutured. The mice were returned to a heated blanket to recover from surgery. In addition, postoperative care was provided, including daily subcutaneous injections of antibiotics for 1 week to prevent bladder or wound infections. We checked the mice’s health twice daily for the entire experiment. Urine was expressed by manual abdominal pressure twice per day until the mice regained reflex bladder function.

### 2.4. Characterizations

A digital camera (Canon EOS 70D) was used to take the optical photographs and videos in this study. For evaluation and analysis of locomotor function in mice, we used two types of Plexiglas boxes. We used a Plexiglas runway (50 cm long, 10 cm wide, 10 cm above ground) to assess over-ground locomotion. For the swimming test, we used another Plexiglas box (60 cm long, 7 cm wide, 20 cm tall), filled with 15 cm of water at a temperature of 23 °C. The morphologies and microstructures of the electrodes and pressure sensors were analyzed by using a scanning electron microscope (Hitachi S-4800, 5 kV). The resistance of the pressure sensor was measured by a LabVIEW-controlled digital source meter (Keithley 2602A), and the test voltage was held constant at 1 V.

## 3. Results 

### 3.1. Structure and Morphology Characterization of the Flexible Pressure Sensors

**Figure 2:**
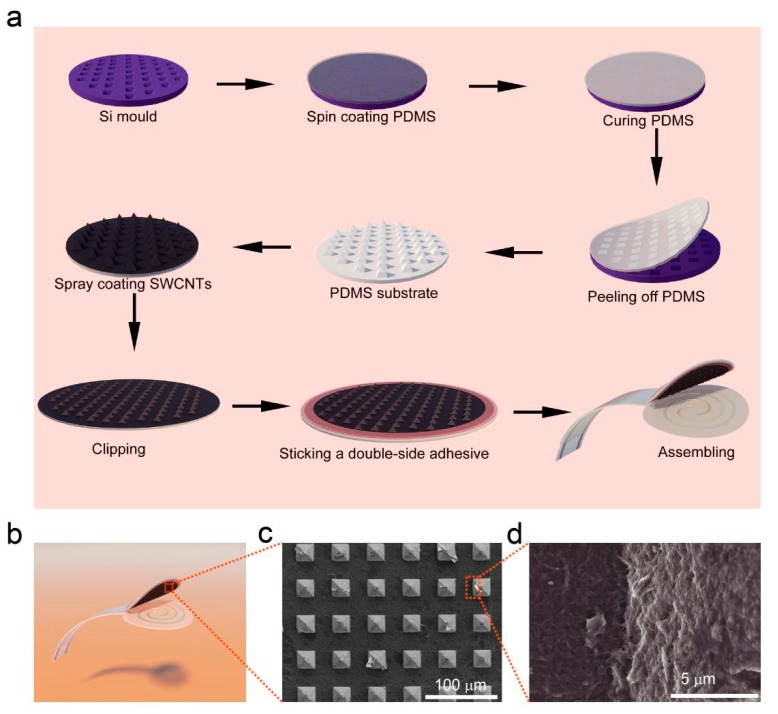
Fabrication and structure of the flexible pressure sensor. (**a**) Schematic of fabrication process for preparing pressure sensors. (**b**) Schematic of the prepared pressure sensor. (**c**) Top-view SEM image of the patterned Polydimethylsiloxane (PDMS)-coated single-walled carbon nanotubes (SWCNTs). (**d**) High-magnification SEM image of the patterned PDMS-coated SWCNTs.

**Figure 3:**
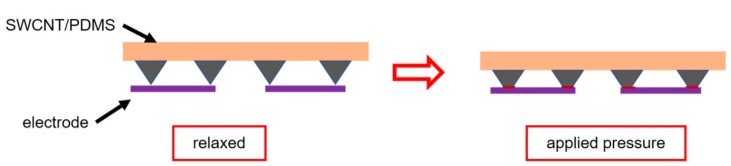
Schematic diagram of sensing mechanism.

Figure 2a illustrates the fabrication procedure for the flexible pressure sensor, and the detailed fabrication process is described in the Experiment section. As schematically shown in Figure 2a,b, the pressure sensor is constructed by assembling a single-walled carbon nanotube/polydimethylsiloxane (SWCNT/PDMS) conductive film with a pyramid microstructure using a sealant onto a thin PET substrate with double-helix Ag electrodes prepared by screen printing. For pressure sensors with such a laminated structure, the mechanism is mainly based on the principle that the contact resistance between the conductive film (SWCNT/PDMS film) and the electrode decreases as the pressure increases (Figure 3) [20]. Obviously, introducing microstructure is an effective way to increase the sensitivity of the sensor. A matrix of pyramidal microunits (dimensions of 25 μm × 25 μm × 15 μm, Figure 2c) was designed to enhance the sensitivity to pressure. SWCNTs were used as the conductive network layer because of their high flexibility, conductivity, and stability. To ensure that the SWCNTs and the PDMS substrate combined effectively, we placed the SWCNT/PDMS film at 200 °C in an air atmosphere to anneal for 30 min. Figure 2d shows that the SWCNTs were partially coated with PDMS due to the thermal cracking of PDMS during annealing, indicating that the components were well combined.

### 3.2. Sensing Performance of the Flexible Pressure Sensors

**Figure 4:**
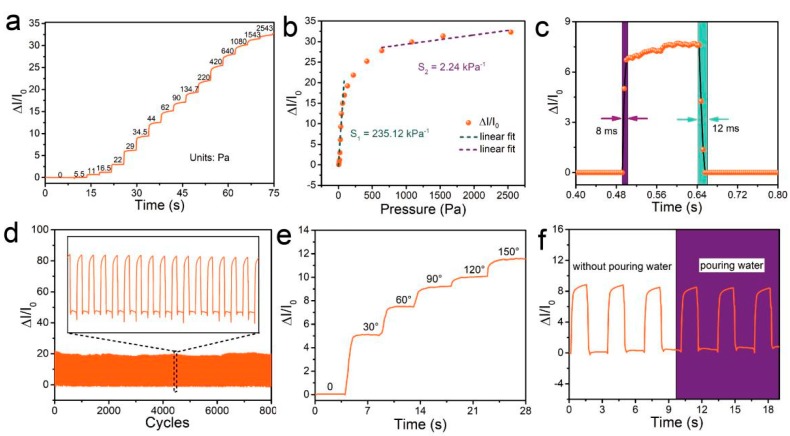
Sensing performance of the pressure sensor. (**a**) Real-time response of the pressure sensor to different degrees of pressure. The applied pressure was increased stepwise using a commercial force gauge (Aliyiqi HF-2N) stepper machine system. (**b**) Relative current change as a function of pressure and linear fitting. (**c**) Time response of the sensor upon applying a quasi-transient step pressure from 0 to 29 Pa. (**d**) Relative current variation under cyclic pressure from 0 Pa to 134 Pa with a frequency of 0.625 Hz over 8000 cycles. (**e**) Relative current change as a function of the bending angle. (**f**) Real-time variation in the relative current. The data were obtained at a stable temperature of about 23 °C.

To confirm the high sensitivity, fast response time, stability, and reproducible readings of the designed pressure sensor, we performed pressure-sensing tests. A pressure sensor was subjected to a force that gradually increased in steps. The variation in the relative current change with respect to pressure is shown in Figure 4a. The relative current change (∆*I*/*I*_0_) was calculated as

Δ*I*/*I*_0_ = (*I* − *I*_0_)/*I*_0_

where *I*_0_ is the sensor current in the relaxed state (Here, *I*_0_ is about 23 μA) and I is the real-time current, measured during the application of pressure. ∆*I*/*I*_0_ increased monotonically to 32 as the pressure was increased to 25.43 kPa. Gauge factors (GFs), the representative parameters to assess pressure sensitivity, were calculated (∆*I*/*I*_0_/*P*, where *P* is the applied pressure, *P* = *F*/*A*, where *F* is the force applied on top of the pressure sensor and *A* is the area over which the pressure is applied) to be 235.12 kPa^−1^ (5.5 ≤ *P* ≤ 134.7 Pa) and 2.24 kPa^−1^ (0.64 ≤ *P* ≤ 25 kPa), as shown in Figure 4b, which is high enough for the pressure sensor to be used for full-range, real-time detection of mouse behaviors. The sensor also exhibits a response time of only 8 ms and a recovery time of 12 ms, which makes it applicable to the detection of animal activities at different speeds (Figure 4c). To verify the reproducibility and stability of measurements made by the sensor, we subjected the device to repeated loading and unloading at an applied pressure of 134 Pa for more than 8000 cycles (1.6 s for each cycle, shown in Figure 4d). The measured value of ∆*I*/*I*_0_ varied periodically, and the signal barely changed between each cycle, which indicates the high durability of the sensor and the reproducibility of its readings. The pressure sensor also exhibited a broad sensing range up to a bending angle of 150° (Figure 4e), with a large accompanying relative current change of ∆*I*/*I*_0_ > 11.5, making the sensor suitable for quantitatively measuring the bending angles of mouse joints. Moreover, the SWCNT/PDMS conductive film was assembled onto a thin PET substrate using a sealant, which endowed the pressure sensor with the ability to function underwater. The waterproofness of the sensor was verified by the purling test (under discontinuous water dropping) shown in Figure 4f, in which the response of the sensor to periodic pressure barely changes.

### 3.3. Selection of Animal Models and Target Muscles

**Figure 5:**
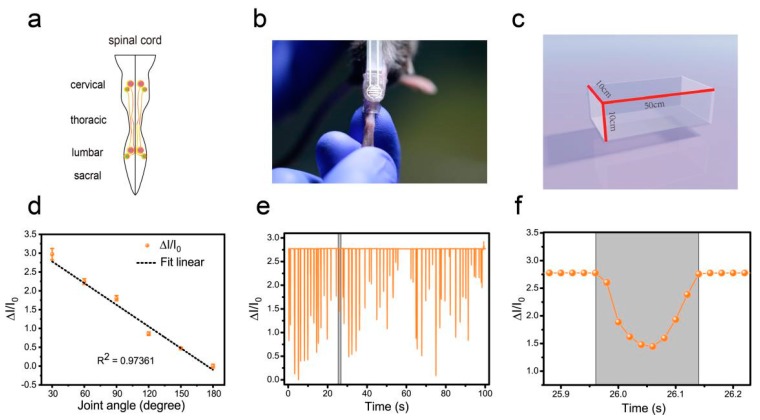
Hindlimb locomotion in open field task (intact mice). (**a**) The pattern of the spinal cord. (**b**) Image of attaching the pressure sensor and the TPE sleeve. (**c**) Plexiglas box for locomotion testing. (**d**) The current rate curve corresponding to different angles (ankle joint). (**e**) Hindlimb locomotion of an intact mouse and its corresponding relative current. (**f**) An enlarged view of one of the actions in part e.

To evaluate motor recovery after SCI in mice and verify the sensitivity of the pressure sensor, we selected the animal model of complete spinal transection [21] and unilateral right-sided thoracic spinal cord injury (extends slightly beyond the midline) [10]. The pattern of the spinal cord was shown in Figure 5a. We compared the recovery of mice at different periods after injury (three days, seven days, and 14 days after SCI). After SCI, the extensors became hyperactive, rendering the flexors unable to contract and causing joint hyperextension. The tibialis anterior (TA) muscle is the most important flexor in the hindlimb of the mouse. Therefore, in this experiment, the TA muscle was selected as the target, and a soft thermoplastic elastomer (TPE) sleeve is designed to comfortably attach the device to the target skin (Figure 5b). Before testing, the hair must be removed from the site. The initial angle of the ankle joint in the intact mice and the SCI mice were 180 degrees (Figure 5b).

### 3.4. Flexible Pressure Sensor was Used to Evaluate the Lower Limb Muscle Movement of Mice in the Open Field Experiment

**Figure 6:**
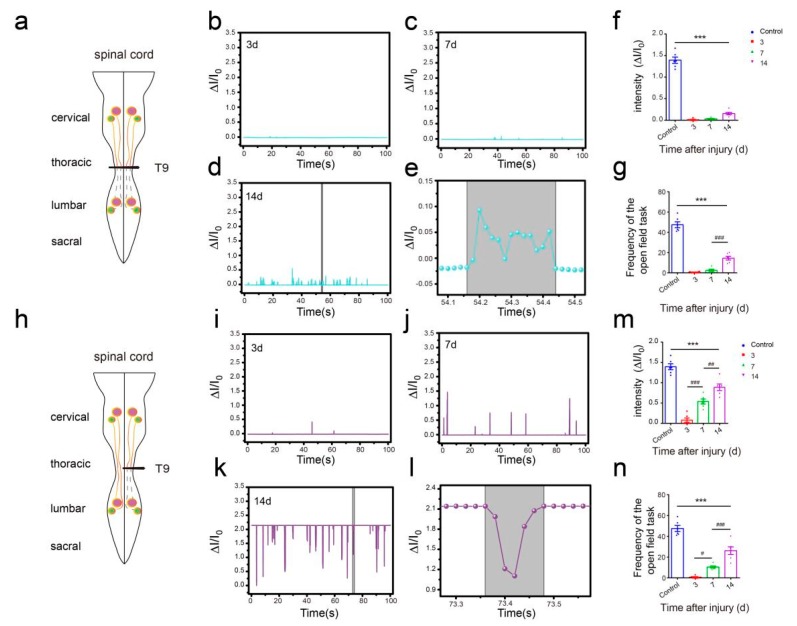
Hindlimb locomotion in open field task (SCI mice). (**a**) The pattern of the spinal cord (complete spinal transection). (**b**) Hindlimb locomotion of complete spinal transection mice and its corresponding relative current (SCI 3d). (**c**) Hindlimb locomotion of complete spinal transection mice and its corresponding relative current (SCI 7d). (**d**) Hindlimb locomotion of complete spinal transection mice and its corresponding relative current (SCI 14d). (**e**) An enlarged view of complete SCI 14d mouse (from Figure 6d). (**f**) Intensity of complete spinal transection mice (*n* = 6). (**g**) Frequency of complete spinal transection mice (*n* = 6). (**h**) The pattern of spinal cord (unilateral thoracic SCI). (**i**) Hindlimb locomotion of unilateral thoracic SCI mice and its corresponding relative current (SCI 3d). (**j**) Hindlimb locomotion of unilateral thoracic SCI mice and its corresponding relative current (SCI 7d). (**k**) Hindlimb locomotion of unilateral thoracic SCI mice and its corresponding relative current (SCI 14d). (**l**) An enlarged view of unilateral thoracic SCI 14d mouse (from Figure 6k). (**m**) Intensity of unilateral thoracic SCI mice (*n* = 6). (**n**) Frequency of unilateral thoracic SCI mice (*n* = 6). One-way repeated-measures AVOVA and post hoc Bonferroni tests were applied. *** *p* < 0.001 (compared with control mice), ^#^ *p* < 0.05, ^##^ *p* < 0.01, ^###^ *p* < 0.001 (compared with SCI 7d mice), error bars, SEM.

**Figure 7:**
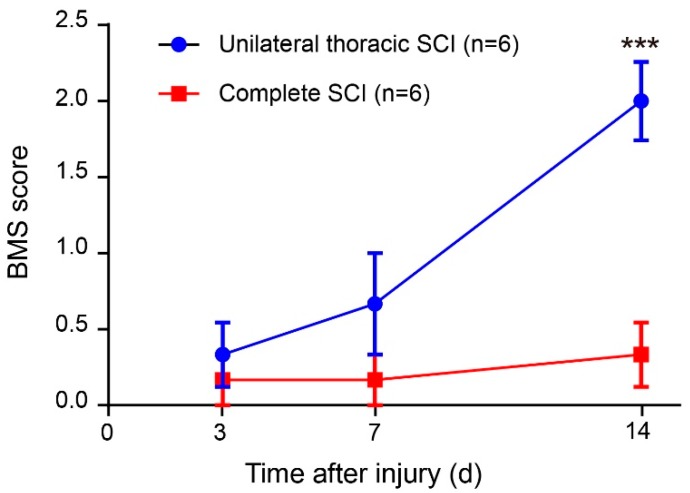
BMS (Basso Mouse Scale) score. Two-way repeated-measures ANOVA followed by post hoc Bonferroni correction. *** *p* < 0.001. Error bars represent SEM.

**Figure 8:**
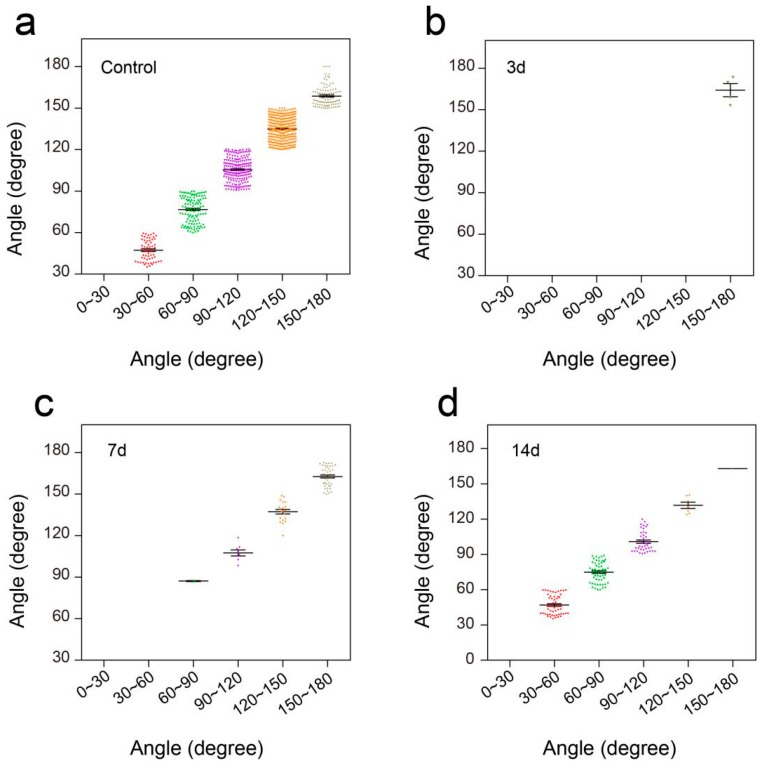
Distribution of ankle angle in open field task. (**a**) Ankle angle distribution of the control group. (**b**) Ankle angle distribution of unilateral thoracic SCI 3d mice. (**c**) Ankle angle distribution of unilateral thoracic SCI 7d mice. (**d**) Ankle angle distribution of unilateral thoracic SCI 14d mice.3.5. Flexible Pressure Sensor was Used to Evaluate the Lower Limb Muscle Movement of Mice in Swimming.

In the open field task, a Plexiglas box (50 cm long, 10 cm wide, 10 cm above ground) was used to assess over-ground locomotion (Figure 5c). The locomotion of the intact mice was not affected after the use of the pressure sensor. Those mice were able to move freely and perform their normal repertoire of actions. Moreover, the pressure sensor fits well and could continuously monitor the locomotion of the animals (Figure 5e). Within two weeks of complete spinal cord transection (The pattern of complete spinal transection was shown in Figure 6a), the mice showed paralysis of the hindlimbs, with almost no movement detected. In the first week of paralysis, almost no sign of movement was detected (Figure 6b,c), no significant difference was observed on the statistical chart (Figure 6f,g). While in the second week, the spastic activity of the lower extremity muscles increased (Figure 6d). The intensity of this muscle spasm was so slight that it was almost invisible in the BMS (Basso Mouse Scale) score (Figure 7), but we can see obvious differences in the statistical chart (Figure 6f,g). A slight activity of the hindlimb was observed three days after spinal cord hemisection injury (Figure 6i, the pattern of unilateral thoracic SCI was shown in Figure 6h), and this activity increased gradually with the prolongation of time in the week after injury (Figure 6j). It was reflected not only in activity intensity but also in activity frequency, we could see the significant difference between the three-day group and the seven-day group of spinal cord injury (Figure 6m,n), but this short-term difference was not reflected in the traditional BMS scoring system (Figure 7). However, the hindlimbs were still not strong enough to support the body. However, in the second week after injury, functional recovery of the hindlimb was observed, which could not only support the body weight but also complete the muscle contraction (Figure 6k). In particular, the activity frequency was sifnificantly higher than that of the seven-day group after SCI (Figure 6n). As shown in Figure 5e and Figure 6d,k, each movement made by a mouse were detected by the pressure sensor to generate a waveform in real time. By selecting and zooming in on waveforms from Figure 5e and Figure 6d,k for analysis (Figure 5f and Figure 6e,l), we found the entire waveform from the intact mice was relatively continuous, and the complete action was carried out (Figure 5f). However, in the 14-day post-complete SCI mice, although the occurrence of a somewhat continuous movement could be recorded, the continuity of the whole movement was observed to be poor, consisting of a series of small movements (Figure 6e). Thus, after the complete spinal cord injury, the flexible pressure sensor recorded a series of muscle spasms. However, the entire waveform from the 14-day post-unilateral thoracic SCI mice was relatively continuous, which was essentially similar to that of an intact mouse (Figure 6l). We used the current rate curve corresponding to different angles to determine the angle corresponding to the current for each action of the intact mice and the unilateral thoracic SCI mice (Figure 5d). The ankle joint angles of the intact mice were distributed evenly between 30 and 180 degrees (Figure 8a). The angle of ankle movement ranged from 60 to 180 degrees within a week of unilateral thoracic SCI (Figure 8b,c). Two weeks after the injury, the angular range of ankle motion increased to 30 to 180 degrees (Figure 8d).

**Figure 9:**
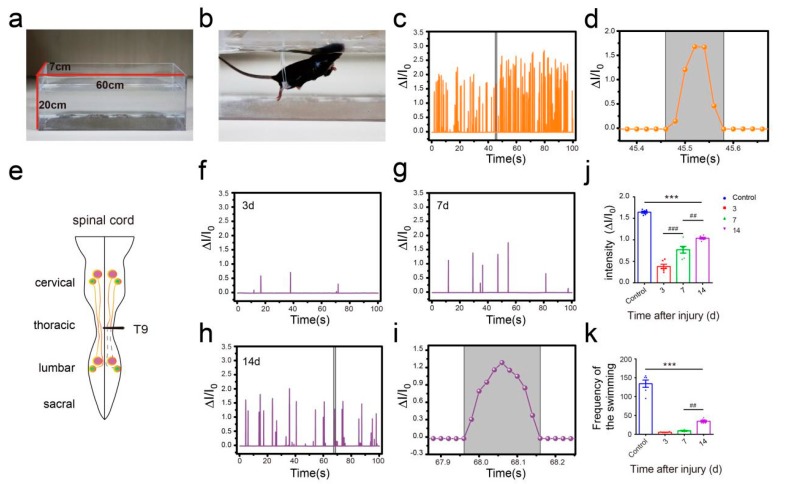
Hindlimb locomotion during swimming. (**a**) Plexiglas box for swimming. (**b**) intact mouse swimming. (**c**) Hindlimb locomotion of intact mouse and its corresponding relative current. (**d**) An enlarged view of one of the actions in part c. (**e**) The pattern of the spinal cord (unilateral thoracic SCI). (**f**) Hindlimb locomotion of unilateral thoracic SCI mice and its corresponding relative current (SCI 3d). (**g**) Hindlimb locomotion of unilateral thoracic SCI mice and its corresponding relative current (SCI 7d). (**h**) Hindlimb locomotion of unilateral thoracic SCI mice and its corresponding relative current (SCI 14d). (**i**) An enlarged view of unilateral thoracic SCI 14d mouse (from Figure 9h). (**j**) Intensity of unilateral thoracic SCI mice (*n* = 6). (**k**) Frequency of unilateral thoracic SCI mice (*n* = 6). One-way repeated-measures AVOVA and post hoc Bonferroni tests were applied. *** *p* < 0.001 (compared with control mice), ^##^ *p* < 0.01, ^###^ *p* < 0.001 (compared with SCI 7d mice), error bars, SEM.

**Figure 10:**
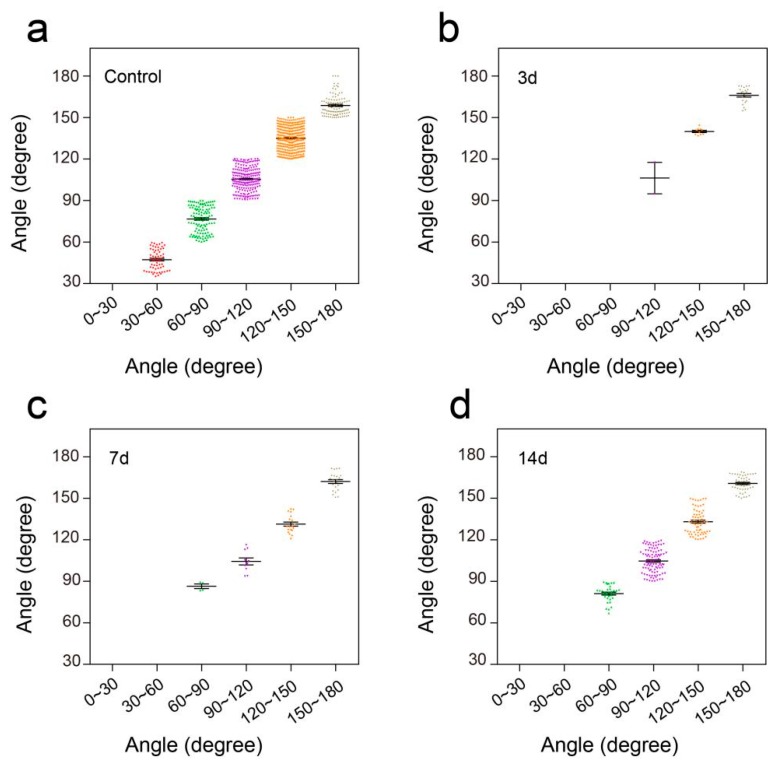
Distribution of ankle angle in swimming. (**a**) Ankle angle distribution of the control group. (**b**) Ankle angle distribution of unilateral thoracic SCI 3d mice. (**c**) Ankle angle distribution of unilateral thoracic SCI 7d mice. (**d**) Ankle angle distribution of unilateral thoracic SCI 14d mice.

To further verify the performance of this pressure sensor under adverse environmental conditions, especially underwater, we conducted a swimming test in intact mice and unilateral thoracic SCI mice (the pattern of unilateral thoracic SCI was shown in Figure 9e). By training the mice to swim in another Plexiglas box (60 cm long, 7 cm wide, 20 cm tall, Figure 9a), we found that the pressure sensor worked well in the water and recorded clear signals, and the mice were able to move around without being affected (Figure 9b). The recorded curve showed that the movement intensity and frequency of the mice in the swimming test were significantly higher than that of the mice in the open-field experiment (Figure 9c). By selecting and zooming in on waveforms from Figure 9c for analysis (Figure 9d), we found the entire waveform from the intact mice was relatively continuous, and the complete action was carried out. As in the open-field experiment, we also quantitatively evaluated the functional recovery of hindlimbs at different periods of unilateral thoracic SCI. It was found that after one week of SCI, the function of hindlimbs recovered significantly in intensity (Figure 9f,g,j). Two weeks later, compared with that of the mice one week after spinal cord injury, the frequency of activity also significantly increased, besides the intensity of activity (Figure 9h,j,k). By selecting and zooming in on waveforms from Figure 9h for analysis (Figure 9i), we found the entire waveform from the mice was relatively continuous. However, the experiment showed that the mice lost balance because of unilateral injury (data not shown). The range of the ankle movement of the intact mice evenly distributed between 30 to 180 degrees (Figure 10a). Three days after SCI, the range of ankle movement was 90 to 180 degrees (Figure 10b). Seven days after SCI, the range of ankle movement became 60 to 180 degrees (Figure 10c). However, the angle of motion did not change significantly after two weeks of injury, similar to that at one week of injury (Figure 10d).

## 4. Discussion

By introducing the pyramid microstructure, we constructed a flexible pressure sensor with high sensing performance of high sensitivity (GF = 235.12 kPa^−1^, 5.5 ≤ *P* ≤ 134.7 Pa), fast response and recover time (8 ms and 12 ms respectively), which can be attributed to the enhancement of pyramid microstructure on the sensitivity, low hysteresis [22,23,24]. Moreover, waterproof design endows the pressure sensor with the ability to function underwater. These advantages ensure that the highly sensitive microstructure-based flexible pressure sensor can be used as a noninvasive device to detect muscle movement in free-moving mice in real-time. 

Effective long-term monitoring of hindlimb disorders caused by central nervous system (CNS) injuries was achieved. After complete transection, the spinal cord below the injury lost innervation from the brain, thus causing the local nerve circuits to be damaged. Within 14 days after SCI, the hindlimb was in a state of paralysis, unable to move or support the body weight. In this experiment, the intensity and frequency of muscle spasm in mice could be detected by using the flexible pressure sensor. It could be seen from the results that the spasticity of the lower limbs of the mice would gradually increase after a week of complete spinal cord injury. However, the intensity was far below the normal level, so the traditional BMS scoring method basically could not see the difference. After unilateral thoracic SCI, using a flexible pressure sensor, we quantitatively evaluated the recovery of hindlimb muscle function in mice at each time period. It could be found that the hindlimbs of the mice recovered to some extent without being rehabilitated, although these mice did not achieve the same motor performance as the intact mice. About a week after spinal cord injury, the lower limb activity of mice began to recover, especially in the intensity of movement. However, the traditional BMS scoring system did not see the difference and the three-day comparison of injury. This reflected the objectivity of the pressure sensor device, which could more accurately reflect the recovery of lower limb motor function in the early stage of spinal cord injury in mice. After two weeks of spinal cord injury, the mice began to stand on their lower limbs to support their weight. The reason for this observation may be related to CPGs in the lumbar segment of the spinal cord [25,26,27]. Previous research also illustrated the importance of providing appropriate sensory afferent feedback to promote neuroplasticity leading to functional recovery after spinal cord injury [28,29,30]. Sensory feedback was still available from the hindlimbs of the SCI mice. In this test, we also made the first attempt to use a pressure sensor to quantitatively assess the recovery of hindlimb motor function in mice at different periods after SCI by comparing the range of joint motion and the corresponding movement time of the mice. Complete transection of the SCI affects afferent proprioception [1,31,32], leading to loss of movement and incomplete movement after a period of time. Our results also suggested the importance of the introduction of proprioceptive information after SCI for post-injury repair. In addition, our findings reflected, to some extent, that this pressure sensor device was suitable for detecting subtle aspects of motor recovery. Compared with the methods of scoring and gait kinematics, the pressure sensor has superior sensitivity to the early recovery of SCI. This feature facilitates the monitoring of early rehabilitation and conveys a meaningful understanding of the treatment of SCI. 

In the previous paragraph, we discussed the difference between the flexible pressure sensor device and BMS score in detail, illustrating the advantages of the device. However, we can see more advantages. First, compared with biomechanical detection of joint movement, the biomechanical method takes time to mark the joints of every small animal to be detected, and capture the movements of small animals with infrared high-speed cameras, so as to analyze the motion of joints [33]. The method of moving the pressure sensor in this paper does not need complicated equipment, and it is easy to operate. Also, Biomechanical testing of animals after spinal cord injury usually requires expensive specialized equipment such as small animal treadmills and weight reduction devices, so the experiment can be carried out only in a few laboratories [34]. On the other hand, the flexible pressure sensor in this experiment has low relative cost and is widely suitable for laboratories at all levels. Moreover, Biomechanical testing requires animals to follow specific orbits. People also need to pay attention to the location and posture of animals [35]. However, in this experiment, animals can run freely in open field, and only need to familiarize themselves with the environment ahead of time. Compared with EMG, the device is non-invasive. First, it circumvented the possible infectivity of implanted electrodes. Secondly, it did not need to consider the interference of other electrical signals, so that data could be collected and analyzed easily.

It would be useful to gather in-depth data on the suitability of this pressure sensor for use in different environments. In future work, we will also perfect the application of this pressure sensor to detect motor function during different animal behaviors, such as ladder climbing and wading, to expand the quantitative behavioral evaluation of mice or other animals.

## 5. Conclusions

A novel, flexible, microstructure-based pressure sensor was designed and fabricated by assembling a sensitive film with a pyramid microstructure onto a thin PET substrate with double-helix Ag electrodes prepared by screen printing. The flexible pressure sensor shows superior sensitivity, which can record muscle spasms in the early stage of severe spinal cord injury and evaluate the recovery of lower limb motor function in the early stage of injury. Moreover, the flexible pressure sensor is waterproof and adaptable, ensuring that it can be used in different environments for non-invasive, long-term and real-time detection. We believe that this flexible pressure sensor holds great promise in many fields, ranging from surgery to health care and wearable electronics.
